# Overweight in children and its perception by parents: cross-sectional observation in a general pediatric outpatient clinic

**DOI:** 10.1186/s12887-017-0964-z

**Published:** 2017-12-22

**Authors:** Daniela Nemecek, Christian Sebelefsky, Astrid Woditschka, Peter Voitl

**Affiliations:** First Vienna Pediatric Medical Center, Vienna, Austria

**Keywords:** Pediatric obesity, Parental perception, Overweight, Children, Austria

## Abstract

**Background:**

Childhood overweight is a growing problem in industrialized countries. Parents play a major role in the development and the treatment of overweight in their children. A key factor here is the perception of their child’s weight status. As we know of other studies, parental perception of children’s weight status is very poor. This study aimed to determine factors associated with childhood overweight and parental misperception of weight status. The height and weight of children, as reported by parents were compared with measured data.

**Methods:**

The study was conducted at a general pediatric outpatient clinic in Vienna, Austria. A total of 600 children (aged 0–14 years) participated in the study. Collection of data was performed by means of a questionnaire comprising items relating to parental weight and social demographics. The parents were also asked to indicate their children’s weight and height, as well as the estimated weight status. Children were weighed and measured and BMI was calculated, allowing a comparison of estimated values and weight categories with the measured data.

**Results:**

Parental BMI, parental weight and a higher birth weight were identified as factors associated with childhood overweight. No association with the parents’ educational status or citizenship could be proven. We compared parents’ estimations of weight and height of their children with measured data. Here we found, that parental estimated values often differ from measured data. Using only parental estimated data to define weight status leads to misclassifications. It could be seen that parents of overweight children tend to underestimate the weight status of their children, compared to parents of children with normal weight.

**Conclusions:**

Pediatricians should bear in mind that parental assessment often differs from the measured weight of their children. Hence children should be weighed and measured regularly to prevent them from becoming overweight. This is of particular importance in children with higher birth weight and children of overweight parents.

**Trial registration:**

Study was not registered. The study was approved by the Ethic committee of the city of Vienna. (EK 13–146-VK).

**Electronic supplementary material:**

The online version of this article (10.1186/s12887-017-0964-z) contains supplementary material, which is available to authorized users.

## Background

Overweight and obesity in childhood are known to be associated, not only with obesity comorbidities in childhood, but also in adulthood, such as hypertension, Type II diabetes and coronary heart disease [1–5]. Being overweight as a child or even being at the risk of becoming overweight (BMI > 85th percentile) is a predictor for being overweight in adulthood [6, 7]. In addition being overweight in childhood influences future comorbidities and shows higher rates of health problems in childhood itself, it also has a strong impact on a person’s emotional wellbeing. Higher BMI values are related to lower self-esteem [8], a higher risk of depression, conduct disorder and lower academic achievements [9, 10]. Halfon et al. [11] conducted a telephonic survey to determine associations between overweight and mental and physical health conditions. They found that parents of overweight children more often report activity restrictions, school problems and a poorer health status in general.

Several studies have focused on factors associated with childhood overweight and obesity. Maternal risk factors include overweight [12], smoking [13] and caesarean section. [14]. Caesarean section has been identified as showing higher Odds Ratio (OR) of obesity prevalence compared with vaginal delivery. But, when stratified for confounders, such as maternal pregnancy weight, there is no statistically significant association [15, 16].

A crucial risk factor for childhood overweight is parental overweight [14, 17, 18]. The OR is doubled with one obese or overweight parent (OR 2.1) and even tripled (OR 3.7) with two obese or overweight parents [13]. This effect is mainly accounted for by a combination of genetic and behavioral factors, as parents have a strong influence on their children’s eating and drinking habits, as well as on their activity levels [19].

To correctly classify children as overweight, studies suggest the necessity of regular weight and height measurements, as parental estimations often do not correspond with actual values [20–23]. Parents tend to underestimate weight for overweight children. On the contrary, in normal weight children, height is often underestimated, leading to a higher attributed weight category [22].

Most of the studies investigating this topic were conducted in kindergartens, schools or pediatric clinics. In contrast this study was conducted at a general pediatric outpatient clinic, as these institutions provide the opportunity to observe children and adolescents over a longer period of time, thereby allowing a continuous monitoring of their individual development and therefore, play a major role in terms of obesity prevention and treatment. Especially, as in Austria children are regularly checked at pediatric outpatient clinics until the age of five in checkups, which are compulsory for extra subventions from the state. Pediatric outpatient clinics combine children’s regular visits to these institutions with trained pediatric staff, in comparison to pediatric clinics, where children mainly go when they are acute ill and are seen by different doctors at every visit. Pediatric outpatient clinics therefore provide a setting, where children are regularly seen by the same pediatrician, which supports prevention of overweight and increasing parental awareness with regards to their children’s weight status.

To prevent childhood overweight, it is necessary to have information on the prevalence and associated factors, especially in younger children, as earlier prevention may lead to better outcomes. In Austria, the availability of data on children’s weight status in children under 3 years is very poor. This study provides essential data concerning this age group. In addition no study on parental perception was done in Austria before.

### Objectives

Main objectives of this study were:

1. To define socioeconomic (parental education), parental associated (parental weight status, parental BMI) and biological factors (birth weight, caesarian section) associated with childhood overweight.
2. To survey the awareness of parents regarding their children’s overweight in a pediatric outpatient clinic
3. To collect data on childhood overweight, in particular, as little is known on the weight status of children under 3 years in Austria
4. To investigate the occurring bias, whether the parent-reported values on height and weight (BMI) or the measured data is used to define the weight status of children.

## Methods

### Study design and population

The data collection for this cross-sectional observational study was conducted at a general pediatric outpatient clinic in Vienna, Austria, from October 2013 to April 2014. A questionnaire was administered to parents accompanying their children to the outpatient clinic. In addition to this, children were weighed and measured.

All the patients visiting the pediatric outpatient clinic on days when questionnaires were provided were asked to take part in this study. In all 670 patients and their parents were asked to take part in the study among them 70 of them refused to participate without stating any reasons. A total of 600 pediatric patients and their accompanying parent(s) were included in the study. All children aged 0–15 years and their parents (18 years and above) could participate in the study irrespective of the reason for consultation. Excluded were only parents, who did not have enough knowledge of the German language. In order to avoid repeated inclusion of the same child and parent(s), a patient number was assigned to each child-this allowed us to identify patients only in the outpatient clinic. Doubly present data was detected using SPSS 21 and the respective second and following entries were deleted. Questionnaires were given to parents while they were waiting for the appointment.

### Questionnaire and anthropometric measurements

The questionnaire contained items on socioeconomic status (parental citizenship and highest educational qualification); birth weight, mode of delivery (caesarian section, vaginal delivery) or birth complications, preterm birth, number of siblings and how long children have been/were breastfed. Parents were also asked to indicate their own height and weight, they were told to estimate the height and weight of their children. Parents were asked to estimate children’s weight status as “underweight”, “normal weight” or “overweight”. To simplify parental assessment no “obese” category was provided. To compare the estimated with the actual weight status, children were weighed and their height was measured by pediatric staff. The questionnaire was designed for this study and was not validated before the study. Questionnaires were provided in the German language only. An translated version of the questionnaire is provided in Additional file 1.

Children were classified as “underweight”, “normal weight”, “overweight” or “obese” using age- and sex-adjusted BMI percentiles developed by Kronemeyer-Hausschild [24].

Children with a BMI under the 5th percentile were classified as underweight, those with a BMI over the 90th percentile were categorized as overweight, while the cut-off point for obesity was the 97th percentile.

### Ethical standards

The study was approved by the Ethics committee of the city of Vienna (protocol no. EK 13–146-VK). Data collection was performed by means of an anonymous and voluntary questionnaire. Informed consent was obtained from all individual participants included in the study. Parents or other persons accompanying the child and children over 6 years had to sign a declaration of agreement.

### Statistical analysis

We performed data analysis using SPSS 21. We evaluated each of the aforementioned variables descriptively for statistical testing the confidence interval was set to 95% (*p* < 0.05). All data used was tested for normal distribution.

In order to determine variables associated with children’s overweight (recoded into a binary variable, using the 90th percentile as a cutoff for overweight), the t-test (metrical variables) and the X^2^ test (categorical variables) were used. For multivariate analyses of predictors of childhood overweight we used binary logistic regression.

To determine factors associated with the correctness of the parental perception binary logistic regression was used. A new variable was created by stating parental perception to be in accordance with the measured weight status (right/wrong).

We compared the estimated values with the measured data using the Spearman correlation coefficient. Spearman’s coefficient was also calculated for the variables weight and height. The BMI was computed separately using the estimated and actual values. The respective results were then compared using the Spearman correlation coefficient. This was carried out for the entire study population and also specifically for overweight and normal weight children.

Concordance of parental perception and estimation with actual weight status was done with kappa statistics.

## Results

Descriptive data is shown in Tables 1 and 2. In 70.3% of the cases the children’s mothers completed the questionnaire, in 16.3% the father, in 12.2% both mother and father and in 1.2% of the cases other family members (stepmother, grandmother, grandfather).

**Table 1:** Characteristics of the sample (metric variables)

	Minimum	Maximum	Mean	Std. deviation
Birth height (cm)	38.0	59.0	50.8	2.71
Birth weight (g)	1200	5400	3317.4	555.67
BMI father (BMI kg/cm^2^)	16.9	47.6	26.3	3.57
BMI mother (BMI, kg/cm^2^)	15.6	46.61	23.81	4.78
Number of siblings	0	6	0.7	0.81
Measured weight (kg)	6.16	79.3	19.3	9.7
Measured height (cm)	64.0	181.0	106.9	21.46
BMI child (BMI, kg/cm^2^)	10.9	29.1	16.1	2.0
BMI percentile child	0.00	100.00	48.80	29.42
Age (years)	0.25	14.75	4.65	2.95

**Table 2:** Characteristics of the sample, categorical variables

		N	Percent
Weight status (BMI)	underweight	61	10.2
	normal weight	456	76.0
	overweight	64	10.7
	Obese	19	3.2
Parental perception	underweight	47	7.8
	Normal weight	533	89.0
	overweight	19	3.2
Age groups	10–15	42	7.0
	6–10	119	19.8
	3–6	225	37.5
	under 3	214	35.7
Sex	male	316	52.7
	female	284	47.3
Person filling in the questionnaire	mother	422	70.5
	father	98	16.4
	Both (mother & father)	73	12.2
	others	6	1.0
Complications during pregnancy or birth	none	340	56.6
	Caesarian section	174	29.1
	Vacuum-extraction	32	5.4
	infection	3	0.5
	others	49	8.2
Only child	Only child	263	43.9
	At least 1 sibling	336	56.1
Highest degree of school education father	compulsory schooling	23	3.9
	completed apprenticeship	224	37.7
	Higher school certificate	153	25.8
	Academic degree	194	32.7
Highest degree of school education mother	Compulsory schooling	28	4.7
	completed apprenticeship	174	29.2
	Higher school certificate	197	33.1
	Academic degree	197	33.1
Paternal weight status	underweight	4	0.7
	Normal weight	291	50.6
	overweight	280	48.7
Maternal weight status	underweight	64	10.9
	Normal weight	349	59.3
	overweight	176	29.9
Parental citizenship	Both parents austrian	461	77.0
	≥ 1 parents not citizen of Austria	138	23.0

The mean age of the children was 4.66 years (range 0.25–14.75 years, SD 2.95). 214 of the 600 patients were under the age of three.

Of all pediatric patients 47.3% were girls and 52.7% were boys. 10.2% were underweight.

The prevalence of overweight was 10.7% and the proportion of obese children was 3.2%. Only slight differences could be determined between boys and girls, with obesity being slightly more common in boys (3.5% vs. 2.8%) and overweight being more commonly observed in girls (9.8% vs. 11.6%), even if this difference was not statistically significant (X^2^p 0.72). Numbers on overweight and obesity were slightly lower in this study, compared to the Austrian obesity report [25].

When subdivided into age groups (under 3, 3–6, 6–10, 10–15 years) and analyzed separately. 7.9% of the children in the under three years category were classified as underweight, 72% as normal weight, 16.4% as overweight and 3.7% as obese. In children aged 3–6 years, 11.6% were classified as underweight 80% as normal weight, 7.1% as overweight and 1.3% as obese. In 6–10 year-olds, 9.2% were classified as underweight, 78.2% as normal weight, 8.4% as overweight and 4.2% as obese. In 10–15 year-olds, 16.7% were classified as underweight, 69% as normal weight, 7.1% as overweight and 7.1% as obese. Results shown in Table 3. Being overweight is most common in children under three years. Surprisingly, when summarizing overweight and obesity in one category, the highest rates were also found in children less than three years, although obesity was most common in the age group of 10–14 years. This effect of age was however not significant in multivariate analysis (*p* = 0.66).

**Table 3:** Weight status separated by age category

Age	Underweight	Normal weight	Overweight	Obese
0–15 years	10.1%	76.0%	10.7%	3.2%
0-3 years	7.9%	72.0%	16.4%	3.7%
3–6 years	11.6%	80.0%	7.1%	1.3%
6–10 years	9.2%	78.2%	8.4%	4.2%
10–15 years	16.7%	69.1%	7.1%	7.1%

We investigated several socio-demographic and anthropometric parameters to find their association with childhood overweight. Result can be seen in Tables 4 and 5.

**Table 4:** Variables that were tested for association with childhood overweight (X^2^ –test)

		Normal weight	Overweight	OR/RR	p (X^2^)	95% CI	
Sex				1.10	0.72	0.69	1.75
	girls	85.6%	14.4%	1.05		0.83	1.33
	boys	86.7%	13.3%	0.96		0.76	1.20
Caesarian section				1.38	0.24	0.84	2.25
	yes	83.3%	16.7%	1.25		0.90	2.25
	no	87.3%	12.7%	0.90		0.77	1.07
Only child				1.45	0.12	0.91	2.30
	yes	83.7%	16.3%	1.22		0.97	1.53
	no	88.1%	11.9%	0.84		0.66	1.06
Highest degree of school education father					0.67		
	compulsory schooling	82.6%	17.4%				
	completed apprenticeship	84.4%	14.6%				
	Higher school certificate	88.2%	11.8%				
	Academic degree	87.1%	12.9%				
Highest degree of school education mother					0.28		
	compulsory schooling	75.0%	25.0%				
	completed apprenticeship	85.0%	15.0%				
	Higher school certificate	87.8%	12.2%				
	Academic degree	87.3%	12.7%				
Parents being overweight				3.8	<0.01	2.02	6.99
	At least one parent overweight	80.9%	19.1%	1.44		1.28	1.63
	none parent overweight	94.1%	5.9%	0.38		0.23	0.64
Parental citizenship				1.16	0.58	0.68	1.97
	At least one parent no citizen of Austria	84.8%	15.2%	1.12		0.75	1.67
	Both parents Austrian.	86.6%	13.4%	0.97		0.85	1.10

**Table 5:** Variables being tested for association with childhood obesity

	OR	*p*-value	95% CI for Exp(B)	
At least one parent overweight	3.51	<0.001	1.865	6.607
Birth weight	1.001	0.001	1.00	1.00
Paternal education level		0.87		
Maternal education level		0.43		
Sex (male)	0.72	0.20	0.44	1.19
Caesarian section	1.31	0.32	0.77	2.22
Being an only child	1.55	0.10	0.92	2.59
Age	0.98	0.66	0.98	0.90

Results of multivariate analysis (binary logistic regression)

Maternal and paternal overweight could be determined as influencing factors. In the case of overweight children 64.6% of the fathers were overweight, compared to 46.2% in the normal weight category. The same was seen for the maternal weight status, with 47.6% of the mothers being overweight in the overweight category compared to 27.0% in the normal weight category. (X^2^ test: *p* < 0.01 both for maternal and paternal weight status).

Corresponding results were obtained for parental BMI (t-test: *p* < 0.01, both for maternal and paternal BMI. The mean maternal BMI was 23.8 (SD 4.78) and the mean paternal BMI was 26.3 (SD 3.58) for the whole study population. In overweight children the mean maternal BMI was 25.9 (SD 5.42) and the mean parental BMI was 27.7 (SD 4.89), compared to mean maternal BMI of 23.5 (SD 4.58) and mean paternal BMI of 26.1 (SD 3.27) in the normal weight group.

While 62.8% of the whole study population had at least one overweight parent, in 17.5% both parents were overweight. Parental overweight has a significant association with childhood overweight. Children with at least one overweight parent had an almost fourfold risk of being overweight (X^2^ OR 3.8, 95% confidence interval 2.02–6.99, *p* < 0.01).

Conclusive with other studies, children with higher birth weight seem to be more likely to be overweight or obese (*p* < 0.01). In all age categories, the mean birth weight was higher in the overweight group compared to children with normal weight. This was also significant in t-test for age categories 0–15 and 3-6 years. The relevant results can be seen in Table 6.

**Table 6:** Association of birth weight and overweight separated by age category

	Mean birth weight	SD		SD		SE	
Age-category	Normal weight		Overweight		Difference		*p*-value (t-test)
0–15 years	3286 g	554.48	3507 g	527.27	+221 g	91.70	<0.001
10–15 years	3310 g	555.61	3557 g	305.94	+247 g	234.69	0.30
6–10 years	3282 g	571.43	3365 g	656.48	+83 g	161.19	0.61
3–6 years	3200 g	553.50	3615 g	396.67	+415 g	130.16	0.002
<3 years	3385 g	532.75	3502 g	554.61	+117 g	91.69	0.20

We found no significant influence of having siblings on overweight. Overweight seems to be slightly more common in only children, compared to the normal weighted group (13.7% vs 8.3%, p0.09). Whereas, data on obese children differs only slightly. (2.7% in only children vs. 3.6% in children having siblings). No significant association could be found when overweight and obese children were summed up in one group. In only children 16.3% were overweight, in the group having siblings results were similar (11.9%). (X^2^ OR 1.45 p 0.12).

No significant association of parental education level and overweight could be determined in this study. In the normal weight group only 3.7% of fathers had a compulsory education compared to 4.9% in overweight children, 36.9% completed apprenticeship as compared to 42.7% in the overweight group, 26.4% had a high school diploma (Matura) as compared to 22.0% in the overweight group and 33.0% had a university degree as compared to 30.5% in the overweight group. (X^2^ p 0.67). Similar results could be seen for the status of maternal educational status (4.1% had only completed compulsory school vs. 8.5% in the overweight group, 28.8% vs. 31.7% had completed apprenticeship, 33.6% vs. 29.3% had a high school diploma and 33.5% vs. 30.5% had a academic degree). (X^2^ p0.28). Results can be seen in Table 7.

**Table 7:** Parental education level separated by weight status

	Weight status	
	Normal weight	Overweight
Paternal education level		
Compulsory education	3.7%	4.9%
Completed apprenticeship	36.9%	42.7%
High school diploma (Matura)	26.4%	22.0%
University degree	33.0%	30.5%
Maternal education level		
Compulsory education	4.1%	8.5%
Completed apprenticeship	28.8%	31.7%
High school diploma (Matura)	33.6%	30.5%
University degree	33.5%	29.3%

No significant effect of caesarian section could be seen. (X^2^, p 0.24, OR 1.38).

In multivariate tests, using binary logistic regression, only parental overweight and birth weight showed a significant association with pediatric overweight. Results can be seen in Table 5. Children with at least one parent being overweight showed higher risk of being overweight (OR 3.51, *p* < 0.01). Birth weight also showed a significant effect on childhood overweight. (OR 1.001, p0.001). Other variables, such as paternal (p0.87), maternal (p 0.43) education level, sex (OR 0.72, p 0.20), Caesarian section (OR 1.31, p 0.32), being an only child (OR 1.55, p 0.095) or age (OR 0.98, p 0.66) did not show a significant association.

### Parental perception of children’s weight status

**Fig. 1:**
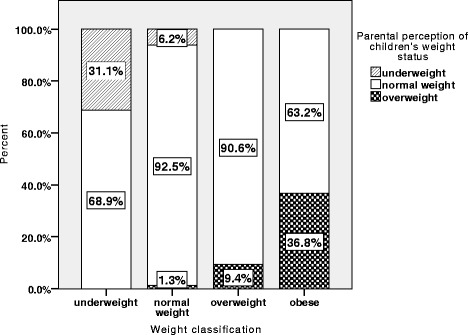
Parental perception of their children’s weight status (Underweight, normal weight, underweight), was compared with weight status according to BMI-percentiles

In the whole study population 75.7% of the children were classified in the correct weight classification by their parents while in underweight children 31.2% were correctly classified as underweight, while the rest was classified as normal weight. In the normal weight category 6.2% were misclassified as being underweight and 1.3% as being overweight. In overweight children only 9.4% were correctly classified as being overweight, while in obese children 36.8% were classified in the overweight category. We provided no obese category for parental estimation of children’s weight status. The relevant data can be seen in Fig. 1.

Comparing parental perception and measured data using kappa statistics, we saw only a fair agreement. (k-value 0.214, *p* < 0.001).

When overweight and obese children summed up in one group, only 15.7% were correctly classified compared to 85.5% in normal weight group. Overweight children therefore are much more likely to be misclassified by their parents. (OR 42.07, *p* < 0.001).

No striking gender differences were seen. Of the obese girls, 37.5% were categorized as being overweight and a similar result was observed boys (36.4%). Only 3.2% of the overweight boys were classified correctly in the overweight category, while this rate was higher for girls (15.2%). All in all, less boys were misperceived by their parents compared to girls, (22.7% vs. 25.7%), because boys are less likely to be misclassified when being underweight. These results were not statistically significant. (OR 0.89, p0.63).

This study found no significant effect of age on parental perception. Only 7.5% of overweight children under 3 years were correctly classified as being overweight, compared to 18.8% in children aged 3–6, 33.3% in children aged 6-10 years and 33.3% in children aged 10–15 years. This effect was not significant in binary logistic regression. (p 0.80).

The only factor that showed a significant effect on the correctness of parental perception in multivariate testing was children’s weight status. Overweight children are more likely to be misclassified by their parents (OR 42.07 *p* < 0.001). Other investigated variables such as age category (p 0.80), paternal weight status (p 0.12), maternal weight status (p 0.28) and child’s sex (OR 0.89 for boys, p 0.63) did not show any significant association with misclassification.

### Data reported by parents vs. measured data

**Fig. 2:**
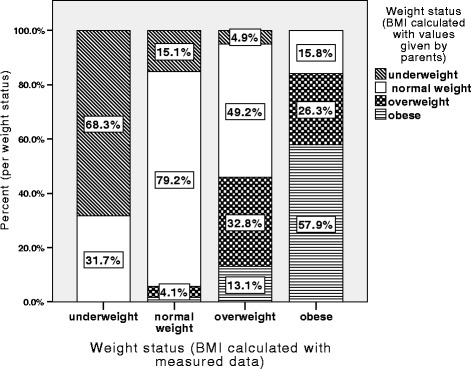
Weight status according to BMI percentiles, calculated using parental estimated weight and height of their children, were compared with data derived from direct measurement by pediatric staff

Furthermore, parents were asked to estimate their children’s current weight and height. We compared these values with values derived from direct measurement by pediatric staff. As can be seen in Fig. 2, proportions of overweight and obesity also differ when only values (weight and height) indicated by parents are used. Especially in overweight and obese children, there was a high risk of misclassification when only data given by parents was used, compared to values measured by pediatric staff. Only 57.9% of the children who were obese would be correctly classified as such, when only parents were asked to name their children’s actual weight and height. The same was seen for overweight children, where a lot of children (54.1%) would be classified as normal- or even underweight.

Spearman’s coefficient was used to compare the data provided by parents with the values measured by pediatric staff. The Spearman correlation coefficient was 0.974 for weight (*p* < 0.001), 0.984 for height (*p* < 0.001) and 0.737 for BMI (*p* < 0.001). This indicates a higher correlation for height and weight compared to BMI. For normal and underweight children the correlation for height was similar to that for overweight children (0.98 vs. 0.98, *p* < 0.001), and it was stronger for weight correlation (0.98 vs. 0.96 *p* < 0.001). The correlation coefficient for BMI in overweight children was lower than that in the whole sample (0.684 vs. 0.737). The comparison between the BMI calculated with values estimated by parents and the measured values indicated a lower accuracy at estimating the BMI of overweight children compared to the BMI of normal weight ones. This led to higher rates of misclassification in overweight children.

Using kappa statistics, it showed that the agreement of weight status comparing measured and parental estimated data, was only a fair agreement. (k-value 0.386, *p* < 0.001).

The person completing the questionnaire (mother and/or father or other persons) had no significant effect on the validity of the estimation of the children’s weight and height. Data not shown.

## Discussion

The number of overweight and obese children is steadily rising [26]. In this study 10.7% of the children were overweight and 3.2% were even obese. Results in this study stated slightly lower numbers of overweight and obesity compared to other national data [25]. This difference may be caused by different type of recruitment, as national data is mainly recruited in kindergartens and schools. This may also be due to the fact that this study includes mainly younger children, only a few were over 10 years and Austrian data shows highest rates of overweight in children over 10 years, even if comparable data does not include children under 3 years. Another reason may be that parents with overweight children may not be as willing to take part in the study. Compared to other data this study shows higher numbers of underweight, especially in children over 10 years this may due to the fact that parents with underweight children are more likely to consult a pediatrician. This study adds data on weight status of Austrian children under 3 years age. According to past research results, overweight children display higher rates of overweight in adulthood [3]. This does not correspond to the common belief of parents that their children might “grow out of their overweight” and that overweight in childhood does not represent a health problem. Most parents do not consult a pediatrician regarding their children’s’ overweight until physical or psychological problems have manifested themselves [19]. This substantiates the major responsibility of pediatricians to promote parental awareness and provide information on the necessity to prevent and treat overweight especially in childhood.

In accordance with other studies, parental overweight and higher birth weight could be determined to have a significant association with childhood obesity [12, 27].

In the current study parental overweight shows a significant association with the children’s weight status, this is in line with former findings [14, 17, 18]. This association was evident for paternal and maternal weight status; it was also identified when using parental BMI in the t-test. Having at least one overweight parent shows a threefold higher risk of being obese as a child in using binary logistic regression. These results substantiate the incontrovertible importance of family based treatments. Integration of families in obesity programs correlates with better long-time outcomes and a greater amount of weight reduction [28, 29].

Identifying factors associated with childhood overweight allows the formation of special target groups. Effective prevention programs could be launched, for younger age groups and those at higher risk of becoming obese. Children with higher birth weight and overweight parents could be identified as target groups, this correlates with former study results [12, 18]. This is particularly interesting in the context of pediatrics, as determinants of adult obesity often go back to childhood.

As already demonstrated in former studies [14, 30], parental awareness regarding children’s overweight is often very poor. We could furnish further proof for the poor parental perception of children’s weight in general. In the current study 63.2% of obese children were classified as being normal weight by their parents. Accordingly, more than 90% of overweight children were classified as normal weight. When investigating the influence of gender on parental perception within this study, we found no significant effect. Overweight boys are slightly more likely to be misjudged by their parents than girls (3.2% vs. 15.1%). This is in accordance with the results of other studies [31–33]. Ideal body images of the Western society might explain the fact that girls are more likely to be correctly classified as overweight. Slim body shapes are commonly regarded as ideal in girls and women, while this perception varies more widely for men and boys. In addition, overweight in men exhibits higher acceptance in society than being overweight as a girl or woman.

The only factor showing a significant influence on correctness of parental perception was children’s weight status.

Compared to other studies, which were done in schools or kindergartens [14, 32, 34], this study has been conducted in a pediatric outpatient clinic. General pediatric institutions are regularly visited by children, especially at a younger age and therefore provide a good setting for overweight and obesity prevention and treatment. No study on parental perception was done before in Austria.

All doctors dealing with young patients and evaluating the weight status should be aware that parents often misperceive the actual height and weight of their children. This is supported by our findings and in line with other authors [20, 21]. Concluding from the current results, many children would be categorized inappropriately regarding their BMI when using values provided by their parents. Using these leads to the falsification of the prevalence of overweight and underweight. The number of overweight children are lower compared with to the data derived from the direct measurement. This relationship inversely applies to underweight children. Correlations between reported data and data from direct measurement were much higher for weight and height (0.974 for weight, 0.964 for height) than for BMI (0.727). These results are similar to those of Huybrechts et al. [35].

The aforementioned findings suggest that children, especially those exposed to higher risks of becoming overweight, should be weighed and measured at regular intervals. This would allow the detection of overweight at an earlier stage and at a younger age, which may lead to more successful prevention programs. Children with higher birth weight or increased parental BMI should be screened for overweight regularly.

### Limitations

Parental weight, height and BMI were only provided by parents and not directly measured. Furthermore, most of the patients attended the outpatient clinic with only one accompanying parent who indicated the weight and height of the other parent. This course of action involves the probability of having a reporting bias.

Parents with overweight children may not be as willing to participate in the study, as overweight and obesity prevalence was lower in this setting compared with other national data [25]. This may have confounded the results.

## Conclusions

Parental overweight and parental BMI could be identified as risk factors for childhood overweight. Children’s birth weight also has an impact on the development of overweight.

This study adds additional weight status data of Austrian children, especially on children aged 3 years and younger.

As in other studies [32, 36, 37], parental perception of their children’s overweight is very poor and only a small number of parents are aware of their children’s actual weight status. Overweight children are misjudged more often, compared with the normal weight children. This misperception could be a major risk factor in the development of overweight and parental education should focus on that as well.This study adds data on parental perception of children’s weight status in Austrian children, as no comparable study has been done before in Austria.

In accordance with other studies [20, 21, 23], this study suggests the necessity of regular measurements of children’s weight and height.

## Additional file

Additional file 1: Questionnaire. (DOCX 72 kb)

## Abbreviations

BMI: Body mass index (weight in kg divided by height in m^2^)
OR: Odds Ratio
