# Supplementary material for: Overweight in children and its perception by parents: cross-sectional observation in a general pediatric outpatient clinic
Source: BMC Pediatr. 2017 Dec 22;17:212. doi: 10.1186/s12887-017-0964-z (PMC5741955; doi:10.1186/s12887-017-0964-z)
Supplement: Additional file 1: — Questionnaire. (DOCX 72 kb) [file 12887_2017_964_MOESM1_ESM.docx]

1. Date:
2. Date of birth of the child _________________
3. Child’s sex
   - male
   - female
4. accompanying parent/ person
   - mother
   - father
   - other_____________
5. Child’s weight _________________kg
6. Birth weight _____________________g
7. Child’s height _________________cm
8. Birth size __________________cm
9. Premature birth
   - yes_____________pregnancy week
   - no
10. Delivery mode
    - spontaneous birth
    - Caeserian section
    - Vacuum extraction
    - Medical induction of labor
    - Infections
    - others_______________
11. Complications during pregnancy
    - None
    - gestationsdiabetes
    - eclampsia
    - HELLP-syndrome
    - Hyperemesis gravidarum
12. Was your child breastfed?
    - yes
      1. how long? ___________month
    - no
13. Does your child suffer of any chronic disease?
    - yes
      1. What kind of diesease?_____________
    - no
14. Father’s weight _______________kg
15. Father’s height __________________cm
16. Estimated father’s weight status is _________
    - normal
    - overweight
    - normalweight
17. Estimated father’s height is _______.
    - normal
    - smaller than average.
    - taller than average
18. Mother’s weight ______________kg
19. Mother’s height________________cm
20. Estimated mother’s weight status is ________.
    - normal
    - overweight
    - underweight
21. Estimated mother’s height is _____.
    - Normal
    - Smaller than average
    - taller than average
22. Maternal citizenship _____________________________
23. Paternal citizenship _____________________________
24. Doe you think your child is underweight, normal weight or overweight?
    - Normal weight
    - overweight
    - underweight
25. Doe you think the child’s heigh is___________
    - normal
    - too small
    - too high
26. Highest degree of school education of the mother
    - compulsory schooling
    - completed apprenticeship
    - Higher school certificate (Matura)
    - Academic degree
27. Highest degree of school education of the father
    - compulsory schooling
    - completed apprenticeship
    - Higher school certificate (Matura)
    - Academic degree
28. Is your child_____
    - firstborn
    - lastborn
    - other ________________
29. Number of siblings_______
30. Does/Did your child go the the kindergarten?
    - yes
    - no
